# Supplementary material for: Influenza A Virus Infection Reactivates Human Endogenous Retroviruses Associated with Modulation of Antiviral Immunity
Source: Viruses. 2022 Jul 21;14(7):1591. doi: 10.3390/v14071591 (PMC9320126; doi:10.3390/v14071591)
Supplement: Supplementary file 1 [file viruses-14-01591-s001.zip › MotifAnalysis.zip!/MotifOutput_SC35MdNS1/homerResults/motif7.similar.html]

motif7

## Information for motif7

C
T
A
G
C
G
T
A
C
T
A
G
C
G
T
A
C
G
T
A
A
G
T
C
A
G
T
C
A
C
G
T
A
C
G
T
A
C
G
T
C
T
G
A
A
C
G
T
  
Reverse Opposite:  

C
G
T
A
A
G
C
T
C
G
T
A
C
G
T
A
C
G
T
A
C
T
A
G
A
C
T
G
A
C
G
T
A
C
G
T
A
G
T
C
C
G
A
T
A
G
T
C
  

|  |  |
| --- | --- |
| p-value: | 1e-51 |
| log p-value: | -1.184e+02 |
| Information Content per bp: | 1.894 |
| Number of Target Sequences with motif | 27.0 |
| Percentage of Target Sequences with motif | 35.06% |
| Number of Background Sequences with motif | 97.7 |
| Percentage of Background Sequences with motif | 0.22% |
| Average Position of motif in Targets | 82.1 +/- 52.0bp |
| Average Position of motif in Background | 85.7 +/- 127.7bp |
| Strand Bias (log2 ratio + to - strand density) | 2.8 |
| Multiplicity (# of sites on avg that occur together) | 1.14 |
| Motif File: | file (matrix) reverse opposite |

### Similar de novo motifs found

|  |  |  |  |  |  |  |  |
| --- | --- | --- | --- | --- | --- | --- | --- |
| Rank | Match Score | Redundant Motif | P-value | log P-value | % of Targets | % of Background | Motif file |
| 1 | 0.729 | A G T C A G T C A C G T A C G T A C G T C G T A A C G T A C T G | 1e-43 | -100.353208 | 49.35% | 1.90% | motif file (matrix) |
| 2 | 0.615 | A T C G T G A C T G C A A T G C A G T C G A C T C A G T A C T G | 1e-32 | -74.775399 | 51.95% | 4.50% | motif file (matrix) |
